# Effect of ultraviolet light treatment on microbiological safety and quality of fresh produce: An overview

**DOI:** 10.3389/fnut.2022.871243

**Published:** 2022-07-22

**Authors:** Veerachandra Yemmireddy, Achyut Adhikari, Juan Moreira

**Affiliations:** ^1^School of Nutrition and Food Sciences, Louisiana State University AgCenter, Baton Rouge, LA, United States; ^2^School of Earth, Environmental and Marine Sciences & Department of Biology, University of Texas Rio Grande Valley, Edinburg, TX, United States

**Keywords:** UV irradiation, fresh produce, pre- and post-harvest contamination, microbiological safety, quality

## Abstract

Fresh and fresh-cut fruits and vegetables have been associated in several foodborne illness outbreaks. Although investigations from those outbreaks reported that the contamination with pathogenic microorganisms may occur at any point in the farm to fork continuum, effective control strategies are still being widely investigated. In that direction, the concept of hurdle technology involving a sequence of different interventions have been widely explored. Among those interventions, ultraviolet (UV) light alone or in combination with other treatments such as use of organic acids or sanitizer solutions, has found to be a promising approach to maintain the microbiological safety and quality of fresh and fresh-cut produce. Recent advances in using UV as a part of hurdle technology on the safety of fresh produce at different stages are presented here. Furthermore, this review discusses the mechanism of UV induced antimicrobial activity, factors that influence antimicrobial efficacy and its effect on produce. In addition, the challenges, and prospects of using UV irradiation as an intervention treatment were also discussed.

## Introduction

Consumer preference toward fresh-like, minimally processed foods with their natural nutritional, sensory and functional properties to prevent or control human diseases has seen meteoric rise over the past decades (1, 2). Minimally processed foods are usually subjected to mild processing or treatment with little to no preservatives (3). Fresh-cut fruits and vegetables are one such example of minimally processed healthful foods. However, fresh and fresh-cut produce have been associated in several foodborne illness outbreaks in recent years. A report by the Center for Science in the Public Interest (CSPI) revealed that fresh produce commodities (17 %) represent the highest number of outbreaks in the United States, during 2002–2011 (4). Between 2010 to 2020, a total of 3,223 foodborne outbreaks with a confirmed food vehicle and etiology occurred in the U.S., of which 13.5% were attributed to fresh produce (5). The available data on the food borne disease outbreak indicates that fresh produce is responsible for the majority of the number of illness and number of illness per outbreak (6).

Studies reported that fresh-cut fruits and vegetables are prone to faster physiological deterioration, biochemical changes and favorable for microbial growth than whole produce (6–8). Fresh-cut processing activities such as washing, peeling, cutting, shredding and/or grating manipulate the intact plant cells to break open and expose intracellular components such as oxidizing enzymes to the outside environment. These conditions accelerate decay (9) decrease the product shelf-life and provides favorable environments for proliferation of microorganisms (10, 11). An analysis of about 1,100 produce-related outbreaks in the United States where a pathogen was identified; majority were caused by bacteria (53%) and viruses (42.5%) and only 4.5% by parasites (12). Thus, it is a challenging task to ensure microbiological safety and quality of fresh produce that are minimally processed and consumed raw. To address these challenges, several chemical and physical interventions have been proposed and implemented with some success.

Most fresh produce packing houses use chemical sanitizers during mechanical washing followed by rinsing with potable water. Sanitizers like chlorine (as sodium or calcium hypochlorite), chlorine dioxide, acidified sodium chlorite, trisodium phosphate, peroxyacetic acid, organic acids (e.g., acetic, lactic, tartaric acid or citric, acetic), electrolyzed water and ozone are often used for this purpose (12). Despite their limited efficacy, some of these approaches are effective in minimizing microbial cross-contamination during washing. More importantly, the efficacy of these compounds depends on various factors like type of produce, target organism, the concentration of sanitizer, treatment time, presence of organic matter, etc. Alternatively, nonthermal and/or nonchemical disinfection technologies such as high-pressure processing, germicidal ultraviolet (UV-C) irradiation, pulsed UV treatment, cold plasma, and ultrasound are gaining increased popularity to reduce food safety risk or to extend the shelf-life of minimally processed foods (13, 14). Among these, germicidal UV treatment has shown promise to enhance microbial safety of fresh and fresh-cut produce at various stages of food production, processing, packaging, storage, and distribution.

Traditionally, UV irradiation has been used for water treatment, surface decontamination, and air disinfection with limited food-related applications (15). However, the use of UV irradiation for applications in the food industry has seen increased interest in the last two decades. Studies have demonstrated UV irradiation's potential to inactivate a wide range of microorganisms (16–18). UV irradiation was proven to be effective against viruses (19), parasites (20) and vegetative cells and fungi (21). Furthermore, UV irradiation was found to reduce the levels of mycotoxins (22) and allergens (23). The disinfection by UV irradiation is a physical method in which the energy is the germicidal medium (24). Minimal effect on quality, absence of residues, and low energy consumption are some advantages of UV irradiation treatment (16, 25). However, poor penetration power, irregular dose delivery, and long treatment times are major limitations of UV treatment (24). In the last decade, extensive research has been conducted in using UV irradiation treatment alone or in combination with other physical and chemical treatments to enhance the safety and quality of minimally processed fresh produce (26–29). However, for the successful application of UV treatment for fresh and fresh-cut produce safety; several important influencing factors need to be considered. In this paper, we present a concise review of the most significant findings on the efficacy of UV treatment alone or in combination with other treatment methods to destroy various foodborne pathogens focusing on fresh and fresh-cut fruits and vegetables. In addition, the effect of UV irradiation treatment on the shelf-life and quality of produce are outlined.

## Principle of UV disinfection

Disinfection is the process of removing bacteria from surfaces. UV irradiation is a part of the electromagnetic spectrum that ranges from 200 to 400 nm. It is mainly subdivided into three regions by wavelength: UV-C (200–280 nm); UV-B (280–320 nm); and UV-A (320–400 nm). UV-C irradiation at a wavelength of about 254 nm has shown to be effective at damaging cells, with the highest DNA absorption indicating UV-C as the most germicidal region (30). The absorption of UV-C irradiation prompts the formation of DNA photoproducts like cyclobutane pyrimidine dimers and pyrimidine 6–4 pyrimidone photoproducts, which obstruct transcription and replication leading to mutagenesis and cell death (16). Low and medium pressure mercury vapor lamps are commonly used as a source of UV irradiation. More details on sources of UV irradiation can be found elsewhere (15). Disinfection efficacy of UV irradiation depends on its fluence or dose delivered. It is defined as the product of intensity (mW/cm^2^) and the exposure time (s) and is commonly expressed as mW-s/cm^2^ or mJ/cm^2^.

## Food applications of UV irradiation treatment

UV irradiation has been used mainly for disinfection of liquid foods and beverages such as milk, juices, ciders, liquid egg, beverages, and honey (15). Also, its application was extended to disinfection of packaging materials, food contact surfaces, in-shell eggs, and surfaces of ready-to-eat meat and meat products (15). Other food processing applications of UV irradiation have been widely discussed (15, 17, 24). Recently, there is a growing body of evidence showing the effectiveness of UV irradiation treatment for the microbial decontamination of irrigation water, fresh and fresh-cut produce as well as process wash waters in the fresh produce industry (31, 32). Some of these studies and their findings were briefly described in the following sections.

### Treatment of irrigation water

Irrigation water is a major conduit of microbial contamination of fresh produce. Treatment of irrigation water with UV irradiation was found to be effective in the disinfection of various plant and environmental pathogens of human health concern. Scarlett et al. (32) compared the efficacy of UV treatment to disinfect several plant pathogens in irrigation water with chlorine and chlorine dioxide treatments. In their study, depending upon the type of plant pathogen, UV irradiation treatment of irrigation water at 250 mJ/cm^2^ and a turbidity of 20 NTU showed higher microbial population reductions than chlorine treatment at 5 ppm concentration. They found that the efficacy of disinfection treatments varied with type of pathogens, time of exposure, flow rate, and type of water. pH-independent disinfection efficacy without forming any known disinfection by-products is a major advantage of UV irradiation over chlorine treatments. Zhang et al. (33) reported that water flow rate, turbidity, organic matter content, the intensity of irradiation and treatment time have significant effects on the disinfection efficacy of UV treatment. Similar observations were also reported by others (34). Sprouts are high-risk food commodities with a history of several foodborne illness outbreaks. The sprouting conditions provide optimal temperature and humidity for any potential pathogens on the seeds or in the irrigation water to grow and survive. UV treatment of water used for sprout production shown to be effective in reducing microbial levels. Ge et al. (35) reported that UV-C irradiation treatment of contaminated irrigation water used for growing mung bean sprouts at 950 mJ/cm^2^ reduced internalized *Salmonella* Typhimurium by 1.84 log CFU/g. They found that the UV irradiation as a pre-harvest intervention significantly decreased *Salmonella* levels in the irrigation water and the internalized organisms in sprouts. Whereas the post-harvest treatment of sprouts with chlorine wash (500, 1,000, or 2,000 mg/L/min), UV treatment (from 78 to 778 mJ/cm^2^) and combined chlorine wash (2,000 mg/L/min) followed by UV irradiation (778 mJ/cm^2^) was found to be ineffective in eliminating internalized pathogens. Moreover, *Salmonellae* were able to recover in the spent irrigation water over a 24-h period and become more resistant to UV irradiation. Adhikari et al. (36) reported that water turbidity can affect the total microbial reduction. *Escherichia coli* in water at turbidity levels as high as 23.32 NTU and treated with UV-C irradiation (20–60 mJ/cm^2^) presented significant reductions. However, as the turbidity decreased to 10.93 NTU the reduction of *E. coli* increased by 2.15 Log MPN, indicating that water quality factors such as turbidity can have a major impact on the effectiveness of UV-C irradiation treatments on irrigation water (36). Studies reported that exposure of bacteria to UV irradiation may cause mutations and increase the UV repair mechanism, thus making the bacteria more resistant to subsequent UV exposures (37). This implies that UV irradiation can be used as a potential pre-harvest intervention to decontaminate irrigation water. Factors such as water type, quality, volume, flow rate, UV intensity, exposure time, and type of organism plays a significant role in the disinfection efficacy.

### Treatment of fresh and fresh-cut produce

Contamination of fresh produce with pathogenic microorganisms during various pre- and post-harvest activities is widely reported. In general, contamination starts at the surface of intact produce and then spreads across interior portions during fresh-cut processing operations. Hence, surface decontamination of fresh produce using chemical sanitizers is a normal practice in the fresh produce industry. However, germicidal UV irradiation can be used as an alternative physical intervention treatment without causing undesirable quality changes and release of toxic disinfection by-products. Several studies have demonstrated that the UV irradiation treatment of fresh and fresh-cut produce is equally if not more efficient in reducing the growth and survival of spoilage and disease-causing organisms than several chemical sanitizers. Kim and Hung (38) found that UV-C irradiation treatment is more effective in reducing *E. coli* O157:H7 on blueberries compared to electrolyzed water and ozone treatments. Levels of *E. coli* O157:H7 were reduced by 1.5 to 2.1 log CFU/g on blueberry calyx and 3.1 to 5.5 log CFU/g on the blueberry skin following application of UV irradiation at 1,200–12,000 mJ/cm^2^. Ozone (4,000 mg/L) and EO water treatments showed only 0.7 log CFU/g on calyx and 0.1 to 1.1 log CFU/g on blueberry skins, respectively (38). Similarly, the UV-C irradiation was more effective in reducing *E. coli* O157:H7 levels on lettuce and apples as compared to 20–320 ppm of chlorine (25). Lower levels of UV-C irradiation treatment at wavelengths between 200 and 280 nm (<100 mJ/cm^2^) were able to achieve similar results on apple surfaces (>2.9 log CFU/g) as compare to ozonated water for 3 min (39), chlorinated water (200 ppm) (40), and ClO_2_ gas treatment at 1.1 mg/L for 10 min (41). Also, treatment of fresh produce with UV irradiation was found to significantly decrease internalized pathogens in lettuce, bean sprouts and other leafy greens (35, 42). Although UV irradiation is proven efficacious for surface decontamination of fresh produce, factors such as produce surface characteristics, UV fluence, method of irradiation delivery, and type and location of organisms were found to play significant role (43). Table 1 provides a summary of selected studies that have demonstrated the antimicrobial efficacy of UV-C irradiation on fresh and fresh-cut fruits and vegetables.

**Table 1:** Studies on UV-C treatment of fresh and fresh-cut fruits and vegetables.

**Produce type**	**Organism(s)**	**UV treatment conditions**	**Log reduction (CFU/g)**	**Light source and wavelength**	**Reference**
Apples	*Escherichia coli* O157:H7	24 mJ/cm^2^	3.3	G36T6 Model 4,136 germicidal light (253.7 nm)	Yaun et al. (25)
	*Escherichia coli* O157:H7	92 mJ/cm^2^ at 23°C	2.9	UV-C Emitter Table-top System (254 nm)	Adhikari et al. (31)
	*Listeria monocytogenes*	375 mJ/cm^2^ at 23°C	1.6	UV-C Emitter Table-top System (254 nm)	Adhikari et al. (31)
	Spoilage Organisms	0.8, 1.2, and 1.6 mJ/cm^2^	1.55 and 2.3	XeMaticA-2 L (180–1,100 nm)	Avalos et al. (44)
Blueberries	*Escherichia coli* O157:H7	1,200–12,000 mJ/cm^2^	1.5 to 2.1 on calyx 3.1 to 5.5 on skin	EF-180 UV system (200–280 nm)	Kim & Hung (38)
	*Salmonella*	0.0105–0.0298 J/cm^2^	3.0 and 4.0	Steripulse-XL RS-3000	Huang et al. (45)
Broccoli (fresh-cut)	*Escherichia coli, S*. Enteritidis, *Listeria monocytogenes*	0 to 1,500 mJ/cm^2^ and storage at 5, 10 and 15°C	1 log at 1.07, 0.02 and 9.26 kJ/m^2^, respectively	15 TUV 36W/G36 T8 Lamps	Martinez-Hernandez et al. (46)
Cantaloupes	*Listeria monocytogenes*	1,190 mJ/cm^2^ at 23°C	1.0	UV-C Emitter Table-top System (254 nm)	Adhikari et al. (31)
Cucumber	*Escherichia coli* K-12	560 mJ/cm^2^ for 6 min followed by 28 days storage at 5°C	1.6	UV-C chamber Reyco Systems (254 nm)	Tarek et al. (43)
Lettuce (leaf)	*Salmonella* spp *Escherichia coli* O157:H7	24 mJ/cm^2^	2.65 to 2.79	G36T6 Model 4,136 germicidal light (253.7 nm)	Yaun et al. (25)
Lettuce (fresh-cut)	*Escherichia coli O157:H7 Salmonella Typhimurium Listeria monocytogenes*	Temperature: 4 and 25°C Illumination distance: 10–50 cm Exposure time: 0.5 to 10 min Exposure zone: One or two sides	1.45, 1.35, 2.12 log at 25°C 0.31, 0.57, 1.16 log at 4°C	5 G6T5 Lamps (254 nm)	Kim et al. (47)
Pears	*Escherichia coli* O157:H7	92 mJ/cm^2^ at 23°C	2.1	UV-C Emitter Table-top System (254 nm)	Adhikari et al. (31)
	*Listeria monocytogenes*	1,190 mJ/cm^2^ at 23°C	1.7	UV-C Emitter Table-top System (254 nm)	Adhikari et al. (31)
Pear (slices)	*Listeria innocua Listeria monocytogenes Escherichia coli Zygosaccharomyces bailli* Mixture of *Zygosaccharomyces bailli, Zygosaccharomyces rouxii, Debaromyces hansenii*	8,700 mJ/cm^2^ for 20 min Slices with and without peel	2.6 to 3.4 log cycles (without peel) 1.8 to 2.5 log cycles (with peel)	TUV-15W G13 T8 55V Lamp System (253.7 nm)	Schnek et al. (48)
Pineapple (sticks)	Spoilage organisms	20 to 480 mJ/cm^2^; Packaged in PET/EVOH/PE trays	Treatment at 200 J/m^2^ then storage at 6°C for up to 15 days showed slower growth of yeast and lactic acid bacteria Counts were 2 log cycles lower than those observed on untreated samples	4 15W/G15 T8 Lamps	Manzocco et al. (49)
Raspberries	*Escherichia coli* O157:H7	1,050 mJ/cm^2^ at 23°C	1.1	UV-C Emitter Table-top System (254 nm)	Adhikari et al. (31)
RTE Salad	*Escherichia coli* O157:H7 *Listeria monocytogenes*	800 mJ/cm^2^ Product placed on SS tray Illumination from both sides at 18 cm away from tray	2.16 to 2.57	15 W, G15T8 Lamps (254 nm)	Chun et al. (50)
Spinach	*Listeria innocua Escherichia coli*	1,000 mJ/cm^2^	1.85 and 1.72	XeMaticA-2L System (180–1,100 nm)	Aguero et al. (51)
Strawberries	*Escherichia coli* O157:H7	720 mJ/cm^2^ at 23°C	2.0	UV-C Emitter Table-top System (254 nm)	Adhikari et al. (31)
	*Listeria monocytogenes*	1,190 mJ/cm^2^ at 23°C	1.0	UV-C Emitter Table-top System (254 nm)	Adhikari et al. (31)
Tomatoes	*Salmonella* spp.	24 mJ/cm^2^	2.19	G36T6 Model 4,136 germicidal light (253.7 nm)	Yaun et al. (25)
Watermelon (fresh-cut)	Spoilage organisms	Packaged fresh-cut watermelons treated at 410 mJ/cm^2^	1	Not specified	Fonseca and Rushing (8)
Zucchini (slices)	Spoilage organisms	10 to 20 min UV-C treatment and storage at 5 or 10°C	Reduced microbial activity and deterioration	15 W, G15T8 Lamps (250–280 nm)	Erkan et al. (52)

### Surface characteristics of fresh produce

Surface characteristics of fresh produce were found to have a significant effect on the disinfection efficacy of UV irradiation treatment (Table 1). Produce with smoother, and even surfaces such as pears, apples and tomatoes were more receptive to UV irradiation (48, 53–55) while rougher or uneven surfaces limit UV exposure for microbial inactivation. Yaun et al. (25) observed the higher effectiveness of UV-C treatment in reducing bacterial populations on the surface of apples than on tomatoes and lettuce. A study by Adhikari et al. (31) reported that UV-C irradiation treatment of organic apples and pears showed a 2.1 to 2.9 log CFU/g reduction of *E. coli* O157:H7 at 92 mJ/cm^2^ whereas strawberries and raspberries required a much higher UV fluency (720 to 1,050 mJ/cm^2^) to achieve only 1.1 to 2 log CFU/g reduction. They found higher inactivation rates on fruits with smoother surfaces such as apples and noticeably lower for fruits that have uneven surfaces, dimples or seeds (strawberries), or druplets (raspberry) that are impermeable to UV-C irradiation (31). In another study by Syamaladevi et al. (56) UV-C treatment at 756 mJ/cm^2^ showed a 3.7 log CFU/g reduction of generic *E. coli* on intact pear surfaces while a 3.1 and 2.91 log CFU/g reductions were observed on wounded pear and peach surfaces, respectively. They concluded that abrasion on the pear and trichomes on peach surfaces protected the microorganisms by shielding from UV-C radiation. Similar results were observed for the inactivation of *Penicillium expansum* on fruit surfaces (56) and inactivation of *E. coli* O157:H7 on blueberry skin and calyx (38).

Manzocco et al. (49) studied the efficacy of UV-C treatment to reduce the microbial load on fresh-cut pineapple sticks. UV-C irradiation did not significantly affect total viable bacteria, yeast and molds. However, the growth of yeast and lactic acid bacteria was slower after UV-C treatment at 20 mJ/cm^2^ and storage at 6°C for up to 15 days. They concluded that the rough surface of pineapple sticks with multiple fruitlets possibly helped microorganisms to avoid UV irradiation exposure. Similarly, Durak et al. (57) reported differences in the surface decontamination efficacy among baby spinach and green onions when subjected to UV-C, acidified sodium hypochlorite (ASC) and a combination of treatments. These differences were mainly attributed to the dissimilarities in surface topographies of each respective fresh produce. They reported that the surface inoculated *E. coli* O157:H7 was likely sheltered and protected from the germicidal effects of UV and ASC treatments on baby spinach. Green onions have smoother surfaces and possess mucus-like compounds that may have helped to interfere with the surface attachment and/or sheltering of the pathogen from UV and ASC treatments.

### UV dose and method of delivery

Several studies reported that the disinfection efficacy of UV irradiation treatment depends on the method of delivery and the dose delivered. Cairns (58) compiled a comprehensive list of lethal UV doses required to achieve different magnitudes of log reduction in various vegetative cells of bacteria (1–7 log), spores (1–4 log), protozoa (1–4 log) and viruses (1–6 log), respectively. Depending on the nature of the organism, the required UV dose or fluency ranged from 0.4 to 235 mJ/cm^2^. It was reported that the degree of cross-linking between thymine and cytosine in the same DNA strand of microbial cells, which is a basis for UV disinfection, is proportional to the amount of UV-C irradiation exposure (59, 60). Allende et al. (10) conducted *in vitro* studies on the inactivation of 20 bacterial strains associated with fresh fruits and vegetables. The UV dose required to completely inhibit the tested strains ranged from 3 to 8.5 mJ/cm^2^. *In vivo* tests in the same study on Red Oak leaf lettuce showed the greatest reductions of natural microflora at a higher dose of 711 mJ/cm^2^. However, treatment at higher doses showed a negative effect on the quality of packaged product upon storage at 5°C for 7 days (10). Another study by Chun et al. (50) reported that the efficacy of UV-C radiation to inactivate *E. coli* O157:H7 and *Listeria monocytogenes* on fresh-cut salad increased with increasing UV dose from 100 to 800 mJ/cm^2^. UV doses of 800 mJ/cm^2^ reduced *E. coli* and *L. monocytogenes* counts on fresh-cut salad by 2.16 and 2.57 log CFU/g, respectively. Fino and Kniel (61) investigated the UV inactivation of three feline calcivirus (a surrogate for norovirus) and two piconavirus (hepatitis A virus and Aichi virus) on green onions, lettuce, and strawberries. They reported a reduction of 1.9–5.6 log TCID_50_/ml on the tested produce and the inactivation of viruses varied depending on the UV dose and the type of produce.

Furthermore, studies reported that the method of UV irradiation delivery onto fruit and vegetable surfaces plays an important role in disinfection. Kim et al. (47) examined the effect of UV-C treatment conditions such as time, intensity, method of exposure, space between sample and UV source, and temperature (4 and 25°C) for inactivating bacterial pathogens such as *E. coli* O157:H7, *Salmonella spp*. and *L. monocytogenes* on fresh-cut lettuce. Treatment at 25°C for 1 min showed a reduction of 1.35 to 2.12 log while at 4°C, only 0.31 to 1.16 log for the tested pathogens. Decreasing the distance between the sample and the lamp to 10 cm and exposing the sample from both sides significantly increased the log reduction (47). Similarly, Lim and Harrison (62) studied the efficacy of UV-C irradiation (0 to 223.1 mJ/cm^2^) to reduce *Salmonella* contamination at various locations on green tomatoes. They reported that regardless of the location of the tomatoes, UV-C treatment was shown to be effective in reducing the levels of *Salmonella*. Liu et al. (63) compared the decontamination efficacy of direct UV exposure with water-assisted UV exposure on blueberries contaminated with *E.coli* O157:H7 or *Salmonella*. They found that water-assisted UV treatment in general showed higher efficacies than direct UV treatment. Method of inoculation affected the inactivation rate with higher reduction (>1.4 log) in blueberries that were spot inoculated than inoculated with dipping technique. As per Fan et al. (64) water assisted, two-sided exposure and tumbling motion during UV-C treatment may minimize the shadowing effect and help increase disinfection efficacy.

More recently, Pulsed UV (PUV) treatment showed promise to reduce microbial populations on the surfaces of fresh produce. Aguero et al. (51) evaluated the efficacy of pulsed UV treatmenton the surface of spinach and reported 1.85 Log CFU/g (*Listeria innocua*) and 1.72 Log CFU/g (*E. coli*) reductions with just two light pulses at fluences lower than 1,000 mJ/cm^2^. However, the authors found that a gradual increase in fluence did not resultedgradual population decrease instead it increased CO_2_ levels and decreased O_2_ in the headspace of treated samples (51). Avalos et al. (44) studied PUV fluences of 0.8, 1.2, and 1.6 mJ/cm^2^ against apple slices and found a 1.55 log CFU/g reduction of mesophilic and psychrophilic bacteria and 2.3 log CFU/g reductions of yeast and mold populations. Another study by Huang et al. (45) tested PUV at 0.0105–0.0298 J/cm^2^ on berries during washing (water turbidity 63.7 NTU) and observed a 3 log CFU/g reduction of *Salmonella* and 4 Log CFU/g when PUV combined with 1% hydrogen peroxide.

### Type of organisms

UV sensitivity of microorganisms varies significantly due to the differences in cellular components such as cell wall structure, thickness, composition, structure of nucleic acid, type of cellular proteins, photoproducts, physiological state of microorganism and the ability of the cell to repair UV damage (15). In addition, the efficacy of UV radiation may vary between species to species, growth media, stage of culture, density of organisms and surface characteristic of the food may also affect (24, 65, 66). Martinez-Hernandez et al. (46) observed high sensitivity of *Salmonella* Enteritidis to UV-C radiation while *L. monocytogenes* was significantly resistant, requiring 2 and 926 mJ/cm^2^ UV doses, respectively when tested on fresh-cut broccoli. Kim et al. (67) studied the bactericidal effect of UVC-LEDs (at four peak wavelengths from 266 to 279 nm) against foodborne pathogens and spoilage microorganisms. They reported that the UV sensitivities of gram-positive, gram-negative bacteria and yeasts differed from each other. For each microorganism groups, higher doses of irradiation resulted in higher reduction levels. Gram-negative organisms showed the lowest resistance while yeasts showed the highest resistance to UVC-LEDs.

UV-C irradiation produces DNA mutations in injured organisms (59, 60). Studies reported that the damage occurred at the DNA level can be repaired by the injured organism when exposed to wavelengths higher than 330 nm (68, 69). Sommer et al. (37) investigated the efficacy of UV-C treatment to disinfect seven pathogenic *E. coli* O157:H7 and one non-pathogenic strain of *E. coli* (ATCC 11229) in water. They found that a UV fluency of up to 30 mJ/cm^2^ is required depending on the strain to achieve a 6-log reduction and that all the strains demonstrated photo repair ability (37). Guerrero-Beltran and Barbosa-Canovas (24) presented a list of photo reactivated microorganisms with higher resistance to UV-C irradiation than non-reactivated microorganisms while Fan et al. (64) discussed the fate of pathogens and potential induction of viable but nonculturable (VBNC) state during post UV-C treatment storage period.

## Combination of treatments

Due to inherent complexity of food matrices and limited penetration depth, the disinfection efficacy of UV irradiation is mostly confined to the surface of the product. Several studies investigated the efficacy of UV irradiation treatment in combination with other treatments to increase overall log reductions (Table 2). UV irradiation combined with laser irradiation was effective against *Bacillus cereus*, compared to UV or laser irradiation alone (71, 72). Durak et al. (57) reported that a combination treatment of UV (125 mJ/cm^2^), acidified sodium hypochlorite (ASC; 200 ppm) and mild heat (50°C) showed more than 5-log reductions of *E. coli* O157:H7 on green onions. While in the same study, a reduction of 2.6 log CFU/g was observed on baby spinach with the combination treatments at 20°C. They concluded that when microorganisms come in contact with produce; depending upon the surface characteristics of the produce, they may infiltrate or internalize, firmly attach to the surface, or become localized into rough surfaces which may protect against the UV radiation. Their results indicate limited effectiveness of individually used UV, ASC, and mild-heat application on both green onions and baby spinach (<3 log) while combination treatments showed a reduction of >5 log on green onions. Hadjok et al. (42) found that fresh produce (such as iceberg lettuce, romaine lettuce, cauliflower florets, baby spinach, sliced Spanish onions, broccoli florets, and ripened whole tomatoes) subjected to a combination of UV-C and H_2_O_2_ treatments yielded higher overall reductions (*E. coli* O157:H7, *Pseudomonas fluorescens, Pectobacterium carotovora*, and *Salmonella*) compared to individual treatments. For example, *Salmonella* counts on lettuce were reduced by 4.12 log CFU with 1.5 % H_2_O_2_ at 50°C and 37.8 mJ/cm^2^ UV fluency while the individual treatments showed only around 2 log reductions (42). In another study by Kim et al. (73) A reduction of 1.8–2.8 log CFU/g bacterial pathogens was achieved on iceberg lettuce by photocatalytic disinfection using TiO_2_ and UV-C irradiation while treatment with UV alone and NaOCl resulted only 1.4 and 1.1 log reductions, respectively.

**Table 2:** UV light in combination with other treatments.

**Treatment**	**Produce**	**Test organisms**	**Test conditions**	**Major findings**	**Reference**
UV-C and organic acid	Fresh-cut Papaya	*Salmonella enterica* ser. *Poona* *Listeria monocytogenes*	UV-C (0, 96, 288, 576, 864 mJ/cm^2^) and Malic acid [0,0.5,1.0, and 1.5 % (w/v)]	864 mJ/cm^2^ UV-C and 1.5 % mallic acid achieved 5.28 and 3.15 log CFU/g reductions for *Salmonella* and *Listeria*	Raybaudi-Massilia et al. (26)
UV-C, Acidified sodium chlorite (ASC), and mild heat	Green onions Baby spinach	*Escherichia coli* O157:H7	UV-C at 12.5 to 500 mJ/cm^2^ ASC at 10 to 200 ppm Mild heat 20 to 50°C Spot and dip inoculation of produce High (7.2 log CFU per spot) and low inoculum levels (4.3 log CFU per spot)	125 mJ/cm^2^ UV-C and 200 ppm ASC at 50°C showed >5 log reduction of spot inoculated green onions at high inoculum level and below detection limit for low inoculum level A reduction of 2.2 log CFU/g for dip inoculated green onions 125 mJ/cm^2^ UV and 200 ppm ASC at 20°C achieved 2.8 log CFU per spot and 2.6 log CFU/g (for dip inoculated) on baby spinach	Durak et al. (57)
UV-C and H_2_O_2_	Iceberg lettuce Romaine lettuce Baby spinach Cauliflower florets Broccoli florets Sliced onions whole tomatoes	*Escherichia coli* O157:H7 *Pectobacterium carotovora, Pseudomonas fluorescens* *Salmonella*	Variable UV doses H_2_O_2_ spray at 480 ml/min	1.5 % H_2_O_2_ at 50°C and UV dose of 37.8 mJ/cm^2^ showed a 4.12 ± 0.45 log CFU of *Salmonella* on the surface of fresh produce. A reduction of 2.84 ± 0.34 log CFU was achieved for internalized bacteria using combined treatments	Hadjok et al. (42)
UV-C and gamma irradiation	Grape tomatoes	*Escherichia coli O157:H7* *Salmonella enterica*	UV-C (60 mJ/cm^2^) and low-dose gamma irradiation (0.1, 0.25, 0.5, 0.75 kGy)	3.4 ± 0.3, 3.0 ± 0.1 log CFU reduction of *Escherichia coli* O157:H7 and *S. enterica* per tomato with 0.6 kJ/m^2^ UVC and 0.25 kGy irradiation More than 4 and 5 log reductions achieved by combined UVC treatment with 0.5 kGy and 0.75 kGy irradiation	Mukhopadhyay et al. (70)

### Process wash water

Postharvest processing of fresh produce requires extensive amount of water to cool, hydrate, wash, and transport products which are considered as high-risk activities. As such, the quality of water is very important and any contamination in water can lead to produce contamination (74). Furthermore, water can serve as a route of cross-contamination and in absence of proper mitigation techniques in place the extended use of the same processing water may result in the build-up of microbial loads, and reduce the effectiveness of chemical sanitizers used in wash water (75). Selma et al. (11) reported that UV treatment of fresh-cut onion, carrot, escarole, and spinach wash waters for 60 min showed a 4 log CFU/mL reduction of microflora while UV, in combination with ozone treatment, showed 6.6 log CFU/mL reduction. They found that UV treatment itself did not change the physicochemical properties of water, but ozone-UV treatment significantly reduced the turbidity of wash water, which helped to increase the disinfection efficacy. Their study concluded that UV treatment could be used as cost-effective intervention only when the levels of undesirable microbial and chemical components in the wash water are at a minimum (11). Millan-Sango et al. (76) studied the efficacy of UV-C (164 mJ/cm^2^) and ultrasound (US; 26 kHz) treatments alone and in-combination for the disinfection of natural microflora in fresh produce wash water. They found that the combination treatment is most efficient and achieved a reduction of 3.57 log CFU/mL. The energy requirements of US, UV and US+UV were 0.107, 0.040, 0.114 kW/h, respectively and the resultant microbial reduction in relation to the energy spent was 4.15, 21.53, and 8.72 × 10^−6^ CFU/mL/J, respectively (76).

## Effect on the quality

Use of UV irradiation treatment has been incorrectly associated with loss of nutritional value and sensory quality (77). However, studies revealed that pre-storage exposure of fresh produce to UV irradiation was effective in minimizing the development of postharvest diseases (78). Studies showing the effect of UV irradiation treatment on the quality of fresh-cut fruits and vegetables were presented in Table 3. Castagna et al. (79) reported that UV-B treatment of two varieties of tomatoes was found to increase phenolic, flavonoid and flavonol concentrations in both peel and flesh. UV-C irradiation activates several biological processes and increases respiratory rate. Erkan et al. (52) reported increase in respiration rates of squash slices with UV treatment and was correlated with the increase in UV-C intensity. In contrast, Vicente et al. (81) found lower respiration rate on UV-C treated peppers than untreated control fruits. Thus, the effect of UV treatment on the quality of whole and fresh-cut fruits and vegetables should be considered on a case-by-case basis with several influencing factors. Though PUV treatment of packaged spinach showed a reduction of *L. innocua* and *E. coli*, shelf-life of product was reduced due to increased CO_2_ and decreased O_2_ levels in the headspace of the package (51). Mukhopadhyay et al. (70) reported no significant changes in the visual and firmness quality of the spinach upon PUV treatment.

**Table 3:** Effect of UV light treatment on the quality of fresh and fresh-cut fruits and vegetables.

**Produce**	**Test conditions**	**Major quality changes**	**Reference**
Tomatoes	Post-harvest irradiation with UV-B light at 608 mJ/cm^2^ per day for 1 h in a climatic chamber for 10 to 22 days Two varieties of fruits tested	•Increased phenolic, flavonol, and flavonoid concentration in both peel and flesh of fruits harvested at mature green stage •Antioxidant activity increased in the peel independently of harvesting stage	Castagna et al. (79)
Fresh-cut tomato	Influence of UV-C at 320 to 1,920 mJ/cm^2^ on nutritional quality of hydroponically grown tomatoes	•When grown under low EC UV-C light minimized development of microbial populations •Increased phenolic content and delayed degradation of Vitamin-C after 7 days of storage at 4–6°C •No effect of UV-C on color, appearance or lycopene content of fresh-cut tomato	Kim et al. (80)
	Low (2.4/2.8 dS/m) or high (4.9/7.7 dS/m) electrical conductivity solutions were tested in hydroponic systems	•Solution with high electrical conductivity decreased phenolic and vitamic C contents by > 10% in fresh-cut tomatoes. While the vitamic-C and lycopene contents are 30% higher in intact fruits harvested at high EC solutions	
		•Degree of salt stress influenced UV-C treatments of fresh-cut tomatoes	
Fresh-cut red cabbage	UV-C at 100, 300, and 500 mJ/cm^2^ for 50, 150 and 250 s	•15 cyanidin derivatives were observed in UV-C treated samples 4 of them were absent in controls •300 mJ/cm^2^ was found to be optimum UV-C dose for enhancing total anthocyanin content	Wu et al. (27) Zhang et al. (33)
	Stored at 4°C in dark after treatment for 1,4,8, or 12 days	•Gene expression relating to anthocyanin metabolism was affected by UV-C irradiation	
		•Increased antioxidant activity •Decreased L, a* and b* values and turned the color darker and increasingly blue	
Fresh-cut melon	UV-C (254 nm) at 4 mJ/cm^2^ Treatment times 30, 60 and 120 s Storage at 5°C	•Enzymatic activity was significantly lower than untreated samples, especially after 7-days of storage at 5°C •7–12% firmer tissue for UV-C treated samples •Irradiation for 120 s at 4 mJ/cm^2^. S was the most effective treatment in reducing both tissue softening and browning	Chisari et al. (28)
Fresh-cut Chokanan mango	UV-C (254 nm) at 15 cm from lamp for 0, 15, 30 and 60 min	•No change in ascorbic acid content of UV-C treated fruits while heat treatment reduced it •Antioxidant activity increased with UV-C treatment while heat treatment decreased it	George et al. (29)
Josephine Pineapple	Heat treatment 70°C for 0, 5, 10 and 20 min	•Shelf-life extended to a maximum of 15 d following treatments •Microbial count in both fruits reduced by both treatments •UV-C treated fruits most accepted by consumers compared to heat-treated fruits	
Cut apples	UV-C at 1,120 mJ/cm^2^ Cut apples impregnated with calcium salts at atmospheric pressure	•1.3 log to non-detectable levels reduction of natural microflora by UV-C treatment •Microbial growth decreased between 0.7 to 2.6 log cycles during 7-day storage at 5°C when compared to controls •No significant change in color due to UV treatment	Gómez et al. (54)
Fresh-cut apples and pears	UV-A light (390 nm) using LED illuminator 8.748 mJ/cm^2^ at 25°C	•Color change of fresh-cut apples decreased by 60% after 60 min exposure •Browning is controlled without effecting organoleptic properties or nutritional quality	Lante et al. (55)
Phuale pineapple	UV-C at 1,320 mJ/cm^2^ for 10 min; 2,640 mJ/cm^2^ for 20 min, 3,960 mJ/cm^2^ for 30 min Postharvest quality properties were measured at every 7 days up to 28 days after irradiation	•Internal browning significantly reduced during storage at 10°C for 28 days. •Disease incidence decreased with increase in UV-C dose •No significant change in color, total soluble solids, total acidity •Significant increase in total phenolic compounds, total flavonoid, and antioxidant capacity in peel •UV-C treatment enhanced vitamin C content in pulp	Sari et al. (14)
Blueberries	Aq. ClO_2_ and UV-C treatment	•Treatment with 2 mg/L ClO_2_ combined with 4 kJ/m^2^ inhibited increase of respiration rate, weight loss, decay incidence and MDA content, delayed decline of firmness, color, and soluble solids content •Improved total anthocyanin content and enhanced the activities of superoxide dismutase, ascorbate peroxidase and phenylalanine ammonia lyase	Xu et al. (18)
Spinach	UV pulse irradiation 1,000 mJ/cm^2^	•Treatment increased the respiration rate of spinach leaves, leading to increase in CO_2_ and reduction of O_2_ in headspace	Aguero et al. (51)
	UV pulse irradiation 15,750 mJ/cm^2^ along with sanitizer made up of hydrogen peroxide, EDTA and Nisin	•No significant effect in visual quality or in texture of samples	Mukhopadhyay et al. (70)

## Concluding remarks

The present review discussed the application of UV radiation during pre/post-harvest application to maintain the safety of fresh produce. Fresh and fresh-cut fruits and vegetables are prone to microbial contamination during various pre- and post-harvest activities. To ensure the safety of these minimally processed produce for human consumption, effective preventive controls should be introduced at various pre- and post-harvest stages. The newly enacted U.S. Food Safety Modernization Act (FSMA) Produce Safety Rule requires all agricultural water must be safe for its intended use. Critical knowledge gap exists on identifying proper disinfecting technique for agricultural water. Any chemical residues in agricultural water would adversely affects crop production or soil quality. This has increased the potential application of UV irradiation at the preharvest level. At post-harvest level, producers are investigating extensively on technologies that are environmentally friendly and could be applied in combination with other methods. This is in fact because of the growing interest of consumer in fresh produce that receives minimal chemical treatments. The use of UV irradiation on post-harvest processing is limited because of the complexity of food matrices. However, recent studies indicated the potential of using UV irradiation in combination with other methods to get similar or even higher efficacy as compared to chemical sanitizers. UV irradiation being a simple and low-cost approach has shown promise as an efficient surface decontamination technique on fresh produce with smoother surfaces. Future studies, should focus on application of UV radiation as part of hurdle technology with other treatments that has the ability to penetrate the surface of fresh produce to achieve an additive or synergistic effect. The effect of UV treatment on the quality of produce needs to be studied on a case-by-case basis.

## Author contributions

Conceptualization, investigation, resources, data curation and writing—original draft preparation, and writing—review and editing: VY, JM, and AA. Visualization, supervision, project administration: VY and AA. Funding acquisition: AA. All authors have read and agreed to the published version of the manuscript.

## Conflict of interest

The authors declare that the research was conducted in the absence of any commercial or financial relationships that could be construed as a potential conflict of interest.

## Publisher's note

All claims expressed in this article are solely those of the authors and do not necessarily represent those of their affiliated organizations, or those of the publisher, the editors and the reviewers. Any product that may be evaluated in this article, or claim that may be made by its manufacturer, is not guaranteed or endorsed by the publisher.
